# Psychometric Properties of the Choosing Wisely Questions in a Large Psychiatric Sample

**DOI:** 10.1002/mpr.70116

**Published:** 2026-10-26

**Authors:** Johan Bengtsson, Alma Andersson, Natalie Forss Lilja, Isabella Hultgren Farsani, Ellen Lissbrant, Björn Wettermark, Ida Blomqvist

**Affiliations:** ^1^ Department of Medical Sciences Uppsala University Uppsala Sweden; ^2^ Uppsala Academic Hospital Uppsala Sweden; ^3^ Department of Pharmacy Uppsala University Uppsala Sweden; ^4^ Department of Clinical Science, Child‐ and Adolescent Psychiatry Umeå University Umeå Sweden

**Keywords:** choosing wisely, low‐value care, patient‐centred care, shared decision‐making, treatment decision‐making

## Abstract

**Objectives:**

The Choosing Wisely Questions (CWQ) support shared decision‐making and aim to reduce low‐value care, but their psychometric properties remain unclear. This study evaluated the structural validity, reliability, and item characteristics of the Swedish CWQ in a psychiatric sample.

**Methods:**

In this cross‐sectional study, adult patients at a Swedish university psychiatric clinic (early 2025) completed four CWQ items on perceived information about their medication (benefits, risks, alternatives, and no treatment). Structural validity was assessed using exploratory and confirmatory factor analyses. Internal consistency was measured with Cronbach's alpha, and item characteristics were examined using a graded response model.

**Results:**

A total of 666 patients (57.2% women; median age 35.0 years) were included. The majority had multiple ICD F‐diagnoses. EFA supported a one‐factor solution explaining 53.8% of the variance. CFA demonstrated excellent model fit (CFI = 0.998, TLI = 0.995, RMSEA = 0.032, SRMR = 0.015). Internal consistency was good (*α* = 0.81, 95% CI 0.79–0.83). In the item response analysis, all items showed high discrimination (*a* = 1.94–2.72) and ordered thresholds. Patients reported more information on benefits than on risks, alternatives, or no treatment (*p* < 0.001).

**Conclusion:**

The Swedish CWQ show good validity, reliability, and item performance, supporting their use as a unidimensional measure of perceived information in psychiatric shared decision‐making.

## 1 Introduction

The Choosing Wisely (CW) initiative was launched in response to the challenge of medical overuse in health care (Wolfson et al. 2014; Brownlee et al. 2017). The global spread of the campaign has been considerable and low‐value care (Verkerk et al. 2018) is increasingly being recognized (Born et al. 2019). The principles of the campaign are numerous, but one tenet is patient‐centered care (Mead and Bower 2000; Langberg et al. 2019), transparent communication, and shared decision‐making (Charles et al. 1997; Lee et al. 2025).

Within a shared decision‐making context, the hope is that more dialogues between physicians, or other healthcare staff, and the patient include a discussion about which tests, treatments or procedures are actually necessary (Elwyn et al. 2012). This has the potential to diminish one type of low‐value care, namely care that ignores or disregards the patients' wishes or that the patient would not have wanted had he/she been properly informed (Guan et al. 2024).

Clinical guidelines and other top‐down‐based recommendations run the risk of increasing low‐value care if followed slavishly, without consideration of the patients' situation and/or preferences. Indeed, the core of evidence‐based medicine lies within the overlap of best available scientific evidence, clinical expertise, and patient values (Sackett et al. 1996). A thoughtful dialogue with the patient regarding their needs is therefore paramount in the proper practice of evidence‐based medicine, using a bottom‐up approach in clinical decisions.

To promote such a dialogue, many national CW campaigns have adopted a set of questions for patients or caregivers (Ansley 2016). These were originally developed by Consumer Reports—an American non‐profit organization involved in the initial launch of the CW campaign (Wolfson et al. 2014). Within the CW community, they are often referred to as the “Choosing Wisely Questions” (CWQ). Although there are national variations, the original questions are as follows:

1. Do I really need this test or procedure?
2. What are the risks and side effects?
3. Are there simpler, safer options?
4. What happens if I don't do anything?
5. How much does it cost, and will my insurance pay for it?

These questions have been adopted by CW campaigns in the United States (ABIM Foundation 2016) and Australia (Choosing Wisely Australia). Some countries have not adopted Question number 5, for example Norway (Legeforeningen Gjør Kloke Valg), Sweden (Svenska Läkaresällskapet Kloka Kliniska Val 2025) and the United Kingdom (Academy of Medical Royal Colleges). The UK questions are referred to as the BRAN questions, deriving from Benefits, Risks, Alternatives, Nothing.

While much attention has been paid to the most effective strategies for reducing low‐value care (Grimshaw et al. 2020; Raudasoja et al. 2022), there has been comparatively little research on the CWQ per se. Recent studies have evaluated the CWQ in shared decision‐making and health literacy contexts (Muscat et al. 2025, 2023). They have also been used as an intervention (Drury et al. 2022), in qualitative studies (Blake et al. 2022), and in radiology decision‐making settings (Allen et al. 2019). The BRAN questions have been studied in various settings (Lal et al. 2023; Bello et al. 2023; Ferro‐López et al. 2021). There are also other, partly overlapping, initiatives for improving shared decision‐making (Shepherd et al. 2016).

However, psychometric groundwork for the CWQ is lacking. Their content validity has already been framed with regard to their clinical use in patient‐centered care and shared decision‐making. The CWQ are not designed to measure an underlying construct, but if such a construct could be identified it would facilitate further research and ultimately have clinical importance. According to the guidelines in the COnsensus‐based Standards for the selection of health Measurement INstruments (COSMIN, https://www.cosmin.nl), the next steps in a psychometric validation process are to investigate structural validity, internal consistency and criterion validity measures.

Notably, a recent review concluded that training programs improve the shared decision‐making skills of general practitioners, but called for more homogeneity in measurement scales to facilitate further research and education (Jaeken et al. 2024). Shared decision‐making is partly guided by values other than clinical effectiveness, such as ethical and legal aspects, however, evidence‐based proof of positive effects would facilitate implementation (Légaré et al. 2018). It has been demonstrated that disadvantaged groups may benefit more than groups with higher health literacy or socioeconomic status (Durand et al. 2014). Patients with mental disorders are more prevalent in socially disadvantaged populations, raising important questions about the need for more attention to shared decision‐making issues within psychiatry (Fryers et al. 2003; Wilkinson and Pickett 2007; Silva et al. 2016).

Many of the caveats raised using the CWQ in research, such as lack of generalizability, relate to the scarce research on the basic psychometric properties of the CWQ. In order to advance their use in quantitative settings, both reliability and validity measures are needed (Ahmad et al. 2020). A stable, potentially generalizable, psychometric core would facilitate their adoption. The aim of this study was, therefore, to assess the psychometric properties of the Swedish version of the CWQ in a psychiatric sample within a shared decision‐making context, using both classical test theory and item response theory (IRT). Reporting standards from the COSMIN initiative were applied (Mokkink et al. 2024).

## 2 Methods

### 2.1 Study Design and Setting

This was a quantitative cross‐sectional study based on survey responses from patients consulting a psychiatric clinic, combined with data from their electronic medical records.

### 2.2 Participants

Inclusion criteria were 18 years or older and having visited or been admitted to the adult psychiatric clinic at Uppsala University Hospital, Sweden. Exclusion criteria were inability to understand Swedish or the research participant information, and/or inability to complete the informed consent form. Participants were recruited from both outpatient and inpatient care, across sections for psychosis, affective, addiction and neuropsychiatric disorders, as well as the emergency psychiatric ward. Thus, all patients visiting the outpatient clinics or being discharged from the inpatient wards meeting the inclusion/exclusion criteria were eligible, regardless of diagnosis, comorbidity, medication status and/or concomitant illicit drug use.

### 2.3 Data Collection Procedure

Data were collected using a questionnaire available both in paper format and digitally via a QR code posted in the outpatient clinics. Eligible patients were approached in the clinic waiting rooms regarding participation and provided with oral and written information about the study. Those agreeing to participate completed the consent form, followed by the questionnaire. Patients who responded via QR code received equivalent information digitally. Data collection took place over 5 weeks in February and March 2025; the QR codes were kept posted for a further 4 weeks. Inpatient staff were consulted daily to identify patients scheduled for discharge who could be approached regarding participating. However, the logistics did not function as intended since patients had often already left the wards once the study personnel arrived. To assess the proportion of registered patients who participated, data was obtained from the hospital's internal registration system. The aim was to approach all patients who met the inclusion/exclusion criteria during the data collection period. However, it was not practically possible to keep count of the number of patients who declined participation or were not approached. Due to the problems with inpatient recruitment, the data from inpatient wards were not included.

### 2.4 The Questionnaire

The questionnaire consisted of eight questions. The first was a yes/no question regarding whether the patient was currently taking any psychiatric medication. If responding “yes,” the participant was asked to provide the medication's name. When multiple medications were being taken, the participant was asked to either indicate the most important one or complete a separate form for each. Questions two to five were based on the Swedish version of the CWQ (https://forening.sls.se/kkv/patienter). In this setting, they were based on the participants' drug regimens and concerned their perception of the information given about their medication treatment. Specifically, participants were asked if they had received sufficient information about the need for the medication (here referred to as “CWQ1 Benefits”), potential risks and side effects (“CWQ2 Risks”), possible treatment alternatives (“CWQ3 Alternatives”), and the implications of forgoing medication (“CWQ4 Nothing”). The last three questions (number six to eight, q6‐8) addressed the patient's experience of involvement in decisions about their medication treatment; their motivation to continue treatment; and their overall satisfaction with their psychiatric care. All questions except the first were answered on a ten‐point response option scale, from 1 (“yes, absolutely”) to 10 (“no, not at all”). Before further analysis, the numbers were inverted so that 1 is equivalent to (“no, not at all”) and 10 to “yes, absolutely”. Higher values therefore represent greater perception of having received information. The questionnaire was pilot tested in a number of patients at the psychiatric emergency ward.

### 2.5 Data Management and Missing Data

All questionnaires were pseudonymized and transferred to a database, where responses via QR code were also automatically stored. Through the date when the participants completed the questionnaire, and the Swedish personal identification number (Ludvigsson et al. 2009), the questionnaires were linked to electronic medical records to retrieve data regarding gender, age, psychiatric diagnoses and prescribed medications. The time window for this data extraction was one month before and after the date when the questionnaire was completed. If a patient had filled in the questionnaire on multiple occasions, the first entry was used. If the participant did not enter a specific drug name on the questionnaire, the medical record data was used to identify the patient's prescribed medication. If still unclear, that participant's medication was categorized as unknown. If participants provided answers for several drugs, only the first entry was used. Forms referring to non‐psychiatric medications were excluded from the analysis and participants with any missing responses to the CWQ were excluded from the study.

### 2.6 Statistical Analysis

#### 2.6.1 Descriptive Statistics and Differences Between Questions

All results are presented for both the whole sample, and for men and women separately. Differences between patients' responses to the four CWQ were analyzed using the Friedman test. Post hoc pairwise comparisons were conducted using Wilcoxon signed‐rank tests. Effect sizes were reported using Kendall's W. These analyses were performed in JASP (JASP Team 2026).

To evaluate convergent validity, Spearman's rho correlations were examined and interpreted according to established guidelines (Schober et al. 2018). The CWQ total score (sum of CWQ 1–4) was correlated with q6 (involvement with treatment, *n* = 665), q7 (motivation for treatment, *n* = 664) and q8 (satisfaction with care, *n* = 662), respectively.

#### 2.6.2 Classical Test Theory Analysis

All statistics below were performed in R (R Core Team 2026). The sample was randomly split into two equal subsamples (*n* = 333 each), with exploratory factor analyses (EFA) conducted in the first subsample and confirmatory factor (CFA) analyses in the second, in order to avoid performing the analyses on the same sample (Fokkema and Greiff 2017). The Kaiser–Meyer–Olkin (KMO), parallel analysis, and EFA was performed using the psych package in R (Revelle 2026). EFA was based on a polychoric correlation matrix and estimated using weighted least squares (WLS). The WLS estimator utilizes the full weight matrix of the polychoric correlation, hence, appropriate for exploratory analyses. KMO values were interpreted as excellent when exceeding 0.90, adequate when above 0.70, and unacceptable when below 0.50 (Kaiser 1974). Evidence of unidimensionality in EFA was assumed if the first factor explained at least 20% of the total variance and if the ratio of variance explained by the first factor to that of the second factor exceeded four (Bentler 1990). CFA was conducted on the polychoric correlation matrix using the diagonal weighted least squares (DWLS) estimator implemented in the lavaan package (Rosseel 2012). The DWLS estimator is a computationally efficient and stable estimator for ordinal data.

A single‐factor CFA model was tested. Model fit was assessed using established criteria: scaled Comparative Fit Index (CFI) and Tucker–Lewis Index (TLI) values > 0.95, scaled Root Mean Square Error of Approximation (RMSEA) < 0.06, and Standardized Root Mean Square Residual (SRMR) < 0.08. The CFA model was applied to the polychoric correlation matrix using the diagonal weighted least squares (DWLS) estimator implemented in the R package lavaan (Rosseel 2012). Internal consistency reliability was assessed using Cronbach's alpha, where values above 0.7 were considered acceptable, above 0.8 good, and above 0.9 excellent (Cronbach and Warrington 1951).

#### 2.6.3 IRT Analysis

Prior to fitting the IRT model, its key assumptions—unidimensionality, local independence, and monotonicity—were examined. Unidimensionality, defined as the extent to which the scale reflects a single latent construct, was evaluated using a one‐factor CFA as described above. Local independence was examined by evaluating the residual correlations after controlling for the dominant factor in the CFA and residual correlations > 0.20 were considered indicators of local dependence (Reeve et al. 2007). Monotonicity was examined using nonparametric item response theory implemented in the Mokken package in R (van der Ark 2007). The assessment was based on the scalability coefficient h, with values over 0.30 considered acceptable for individual items and values over 0.50 for the overall scale.

We fitted a Graded Response Model (GRM) model using the mirt package in R (Chalmers 2012; Masur 2022). GRMs are suitable for items with ordinal response categories and produce estimates of item slopes and thresholds. The slope parameter reflects an item's discrimination, where higher values indicate a greater ability to differentiate between respondents with different levels of the underlying trait. The threshold parameters represent item difficulty; for a 10‐point response scale, nine thresholds are estimated across the continuum of the measured trait.

We evaluated the fit of the items with Orlando and Thissen's S‐X2 statistics (Orlando and Thissen 2003). A non‐significant value is an indication of adequate fit (*p* > 0.001) (Masur 2022). Measurement precision across levels of the latent trait was examined using the test information function and corresponding standard error of measurement.

### 2.7 Ethics

The study was approved by the Swedish Ethical Review Authority (registration number 2024‐06594‐01) and conducted in accordance with the ethical principles of the Declaration of Helsinki (World Medical 2001). All participants gave informed consent after receiving detailed information about the study's purpose, data protection, and voluntary participation. It was emphasized that participation would not affect the patient's psychiatric care and that consent could be withdrawn at any time without consequences.

## 3 Results

### 3.1 Participants Characteristics

A total of 1973 patients were registered at the clinics during the study period. Of these, 742 filled in the questionnaire (proportion of registered patients who participated 742/1973 = 37.6%); however, 59 did not report any psychiatric drug and 17 were excluded due to missing data and therefore listwise deleted from the analyses, yielding a final sample of 666 participants (proportion 666/1973 = 33.8%). See Figure 1.

**FIGURE 1:**
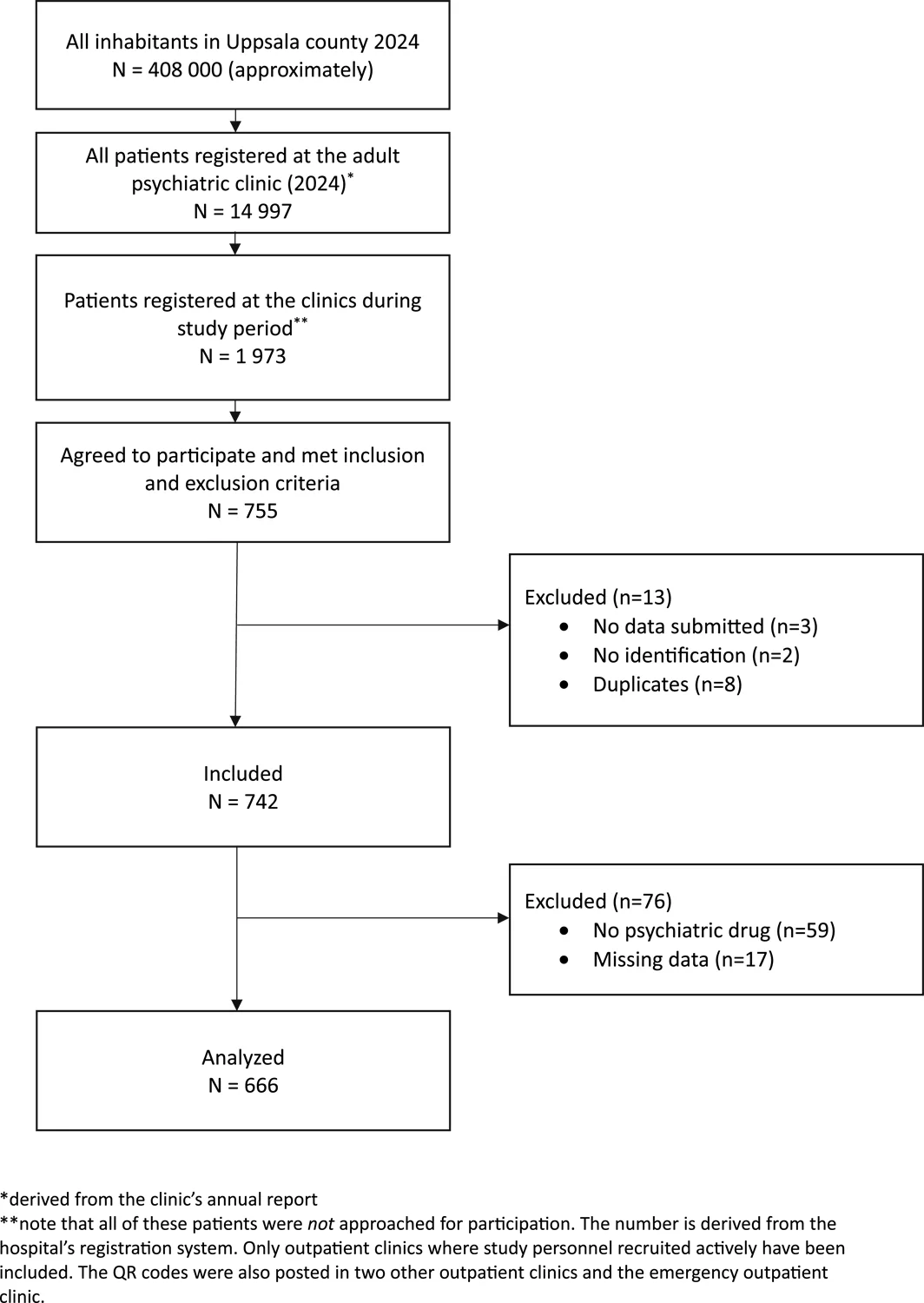
Flow chart of participants.

Of the included participants, 285 (42.8%) were men and 381 (57.2%) women; median age in the total sample was 35.0 years (IQR 20.8). Participant characteristics stratified by gender are presented in Table 1.

**TABLE 1:** Characteristics of the sample.

	Male (*n* = 285)	Female (*n* = 381)	Whole sample (*n* = 666)
Age (mean SD/median IQR)	39.8 (13.2)/36.0 (20.0)	37.9 (13.8)/35.0 (21.0)	38.7 (13.6)/35.0 (20.8)
Diagnoses (*n*, %)^a^			
F00–F09	4 (1.4)	1 (0.3)	5 (0.8)
F10–F19	64 (22.7)	46 (12.2)	110 (16.7)
F20–F29	56 (19.9)	48 (12.7)	104 (15.8)
Of which F20	31 (11.0)	19 (5.0)	50 (7.6)
F30–F31	41 (14.5)	73 (19.4)	114 (17.3)
F32–F33	53 (18.8)	82 (21.8)	135 (20.5)
F34–F39	5 (1.8)	3 (0.8)	8 (1.2)
F40–F48	73 (25.9)	161 (42.7)	234 (35.5)
F50–F59	14 (5.0)	47 (12.5)	61 (9.3)
Of which F50	2 (0.7)	35 (9.3)	37 (5.6)
F60–F69	21 (7.4)	58 (15.4)	79 (12.0)
F70–F79	5 (1.8)	4 (1.1)	9 (1.4)
F80–F89	26 (9.2)	41 (10.9)	67 (10.2)
F90–F98^b^	83 (29.4)	107 (28.4)	190 (28.8)
R‐code	32 (11.3)	43 (11.4)	75 (11.4)
Z‐code	127 (45.0)	164 (43.5)	291 (44.2)
Number of ICD F‐codes^c^			
None	14 (5.0)	23 (6.1)	37 (5.6)
One	125 (44.3)	132 (35.0)	257 (39.0)
Two	66 (23.4)	83 (22.0)	149 (22.6)
Three	34 (12.1)	62 (16.4)	96 (14.6)
Four	27 (9.6)	35 (9.3)	62 (9.4)
Five or more^d^	16 (5.7)	42 (11.1)	58 (8.8)
Drug reported in questionnaire (*n*/%)^e^			
Agent used in addiction	27 (9.5)	12 (3.1)	39 (5.9)
Antipsychotic	67 (23.5)	64 (16.8)	131 (19.7)
Antidepressant	68 (23.9)	123 (32.3)	191 (28.7)
Mood stabilizer	34 (11.9)	59 (15.5)	93 (14.0)
Agent used in ADHD	52 (18.2)	64 (16.8)	116 (17.4)
Tranquilizer	12 (4.2)	29 (7.6)	41 (6.2)
Sedatives	10 (3.5)	10 (2.6)	30 (4.5)
Other	3 (1.1)	8 (2.1)	11 (1.7)
Unknown	12 (4.2)	12 (3.1)	24 (3.6)
Information about… (median, IQR)			
Benefits of medication (CWQ1 *Benefits*)	9 (2)	10 (2)	10 (2)
Risks/side‐effects of medication (CWQ2 *Risks*)	8 (6)	7 (5)	7 (5)
Alternatives to medication (CWQ3 *Alternatives*)	7 (5)	7 (5)	7 (5)
Implications of forgoing medication (CWQ4 *Nothing*)	8 (5)	8 (6)	8 (6)

^a^ Note that one person might have several diagnoses so the sum of percentages could be over 100%. Also note that there were 7 missing responses (3 males and 4 females), which makes the total number 659.

^b^ All of which F90.

^c^ 7 missing (3 males and 4 females).

^d^ Of which five diagnoses (9 men, 19 women), six diagnoses (4 men, 12 women), seven diagnoses (2 men, 4 women), eight diagnoses (1 man, 2 women), nine diagnoses (4 women) and twelve diagnoses (1 woman).

^e^ Agents used in addiction treatment included buprenorphine, buprenorphine + naloxone, disulfiram, methadone, naltrexone, clonidine, and acamprosate. Antipsychotics included paliperidone, aripiprazole, zuclopenthixol, risperidone, quetiapine, olanzapine, clozapine, injectable paliperidone, perphenazine, haloperidol, flupentixol, injectable olanzapine, and lurasidone. Antidepressants included venlafaxine, bupropion, escitalopram, citalopram, sertraline, mirtazapine, fluoxetine, paroxetine, duloxetine, tranylcypromine, vortioxetine, nortriptyline, clomipramine, phenelzine, agomelatine, amitriptyline, and mianserin. Mood stabilizers included lithium, lamotrigine, and valproic acid. Agents used in ADHD included lisdexamfetamine, methylphenidate, dexamfetamine, and atomoxetine. Tranquilizers (anxiolytics) included hydroxyzine, alimemazine, oxazepam, promethazine, diazepam, buspirone, and clonazepam. Hypnotics (sleeping pills) included melatonin, propiomazine, zopiclone, and zolpidem. Other medications included carbamazepine, topiramate, and pregabalin. Note that one person might have several medications so the sum of percentages could be over 100%.

Psychiatric diagnoses covered a broad clinical spectrum. The most common diagnostic groups were anxiety and stress‐related disorders (ICD‐10 F40–F48; 35.5%), mood disorders (F30–F39; 39.0%), neurodevelopmental disorders (F90–F98; 28.9%), substance use disorders (F10–F19; 16.7%), and psychotic disorders (F20–F29; 15.8%).

Multiple psychiatric diagnoses were common: 61.0% of participants had two or more ICD F‐diagnoses, and 8.8% had five or more. Notably, women tended to have more diagnoses than men. In Table 1, all participants with five or more diagnoses (16 men and 42 women) are shown in the same row. Of these, nine men had five diagnoses, after which the numbers declined proportionally. Nineteen women had five diagnoses, twelve had six and eleven had seven or more. The maximum number of diagnoses was eight for men (*n* = 1) and twelve for women (*n* = 1). Diagnostic information was missing for seven participants (1.1%).

The most frequently reported medication classes were antidepressants (28.7%), antipsychotics (19.7%), agents used in ADHD (17.4%), and mood stabilizers (14.0%). Medication could not be determined for 24 participants (3.6%). Agents used in treating addiction were more common in men, whereas antidepressants, mood stabilizers and tranquilizers were more common in women.

Descriptive statistics for the CWQ revealed that, on the whole, participants felt informed about the benefits of their medication (CWQ1 Benefits, median 10), but somewhat less informed about the other CWQ (median 7–8).

Spearman's rho correlations showed evidence for significant positive correlation between CWQ total score and participation (*ρ* = 0.56), motivation (*ρ* = 0.51), and satisfaction (*ρ* = 0.47), *p* < 0.001 for all.

### 3.2 Comparisons Between the Questions

The Friedman test showed a significant difference between questions, χ^2^ (3) = 402.0, *p* < 0.001, Kendall's *W* = 0.20. Post hoc pairwise comparisons revealed that scores for CWQ1 *Benefits* were significantly higher than for all other CWQ. Scores for CWQ2 *Risks* were significantly higher than for CWQ3 *Alternatives*. Lastly, scores for CWQ3 *Alternatives* were significantly lower than for CWQ4 *Nothing*. See Table 2 for post hoc comparisons.

**TABLE 2:** Post hoc comparisons of the differences between the CWQ.

		Mean difference	*t*	p_holm_
CWQ1 *benefits*	CWQ2 *Risks*	1.78	17.3	< 0.001
	CWQ3 *Alternatives*	2.04	18.8	< 0.001
	CWQ4 *Nothing*	1.53	13.8	< 0.001
CWQ2 *risks*	CWQ3 *Alternatives*	0.27	2.6	0.017
	CWQ4 *Nothing*	−0.25	−2.2	0.028
CWQ3 *alternatives*	CWQ4 *Nothing*	−0.52	−4.5	< 0.001

*Note: p*‐value adjusted for comparing a family of 6 estimates.

### 3.3 Classical Test Theory Analysis

In the exploratory factor analysis, sampling adequacy was acceptable (KMO overall measure = 0.79; item‐level values 0.76–0.82). Parallel analysis indicated a one‐factor solution. A single‐factor model extracted using weighted least squares estimation explained 53.8% of the total variance, with all items showing substantial factor loadings ranging from 0.67 to 0.81 (Table 3). The ratio of variance, explained by the first to the second factor exceeding four, supporting unidimensionality.

**TABLE 3:** Exploratory factor analysis of the CWQ.

Item	Factor loading
CWQ1 *benefits*	0.67
CWQ2 *risks*	0.81
CWQ3 *alternatives*	0.73
CWQ4 *nothing*	0.72

*Note:* Eigenvalue (Factor 1): 2.15. Variance explained: 53.8%. Exploratory factor analysis conducted using weighted least squares estimation on polychoric correlations (*n* = 333).

Confirmatory factor analysis of the one‐factor model demonstrated excellent model fit: *χ* ^2^(2) = 2.70, *p* = 0.259; CFI = 0.998; TLI = 0.995; RMSEA = 0.032 (90% CI 0.000–0.119); SRMR = 0.015. Standardized factor loadings ranged from 0.65 to 0.79 (Figure 2). Together, these results support a unidimensional structure of the CWQ. In summary, structural validity of the CWQ was shown through exploratory and confirmatory factor analyses.

**FIGURE 2:**
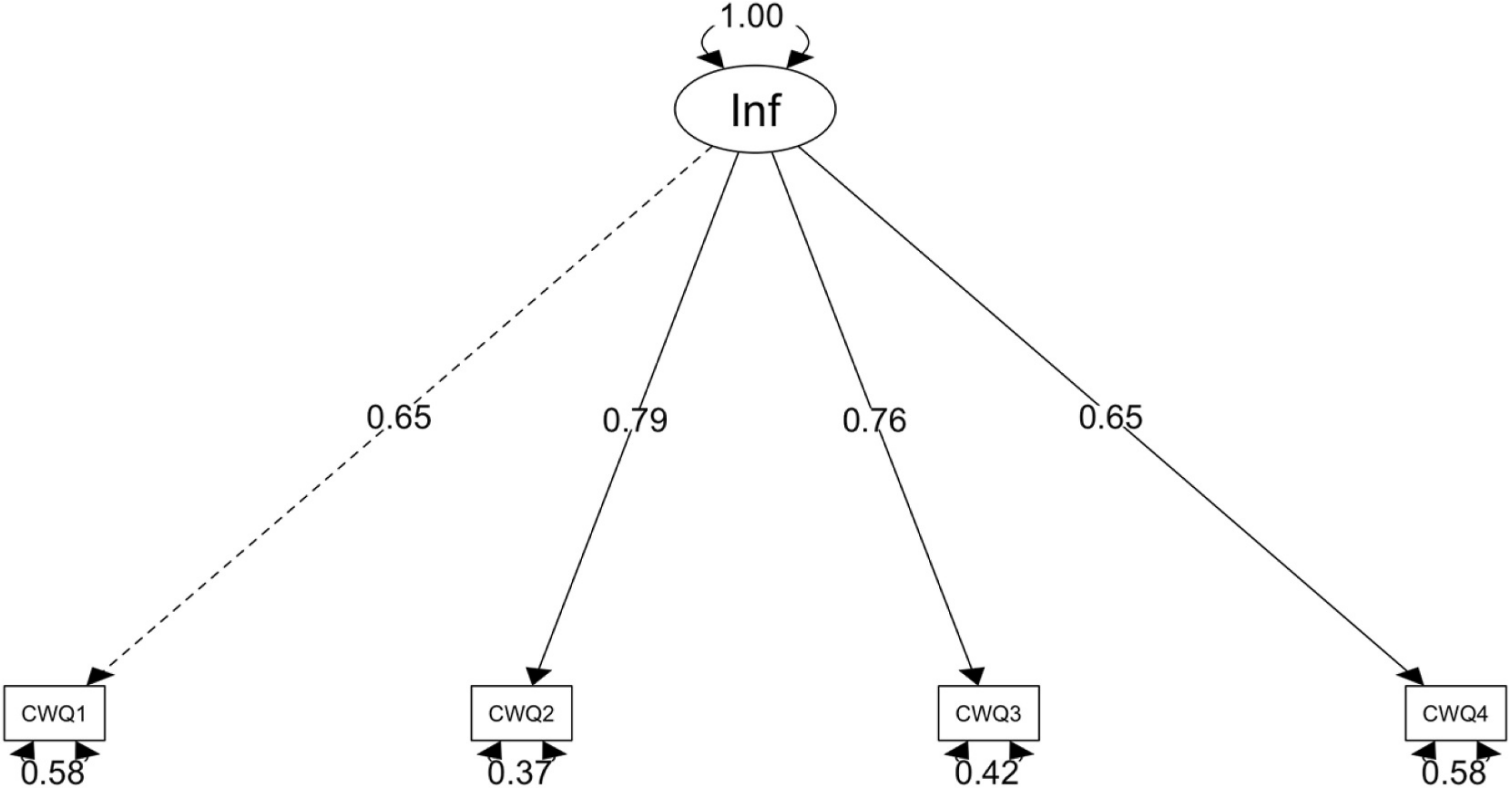
Confirmatory factor analysis of the Choosing Wisely Questions (CWQ). Path diagram of the one‐factor confirmatory factor analysis model for the CWQ (*n* = 333). Standardized factor loadings are shown on the arrows. All loadings were statistically significant (*p* < 0.001), supporting a unidimensional structure.

Internal consistency of the CWQ was good. Cronbach's alpha for the four‐item scale was 0.81 (95% CI 0.79–0.83, *n* = 666).

### 3.4 Item Response Theory Analysis

Item‐level measurement properties were further examined using IRT in accordance with COSMIN recommendations for evaluating item performance and measurement precision. The IRT assumptions of unidimensionality, local independence, and monotonicity were met. First, unidimensionality was supported as shown by for example CFA analysis above. Second, no item pairs had a residual correlation > 0.2, hence local independence was found. Third, all items had a coefficient *h* above 0.3 with a value of 0.55 for the whole scale, hence monotonicity was shown.

A unidimensional graded response model (GRM) was fitted to the four CWQ items.

All items demonstrated high discrimination parameters (*a* = 1.94–2.72), indicating a strong ability to differentiate between respondents at different levels of the latent trait (Table 4).

**TABLE 4:** Graded response model item parameters and fit statistics.

	Item parameters										Item fit statistics			
Item	*a*	*b*_1_	*b*_2_	*b*_3_	*b*_4_	*b*_5_	*b*_6_	*b*_7_	*b*_8_	*b*_9_	*S‐X* ^2^	df	*RMSEA*	*p‐value*
CWQ1 *benefits*	1.94	−2.76	−2.42	−1.95	−1.63	−1.44	−1.13	−0.95	−0.54	−0.02	109.9	90	0.018	0.075
CWQ2 *risks*	2.72	−1.70	−1.48	−1.01	−0.63	−0.45	−0.15	0.05	0–40	0.82	129.8	95	0.023	0.010
CWQ3 *alternatives*	2.47	−1.63	−1.42	−0.96	−0.60	−0.40	−0.05	0.21	0.56	1.01	123.3	99	0.019	0.049
CWQ4 *nothing*	1.95	−1.88	−1.54	−1.13	−0.78	−0.55	−0.27	−0.09	0.28	0.64	114.9	113	0.005	0.432

*Note:* Item fit evaluated using S‐X^2^ statistics.

Threshold parameters were ordered as expected across response categories, supporting appropriate functioning of the ordinal response scale. Item characteristic (trace) curves illustrating category response probabilities across the latent continuum are shown in Figure 3.

**FIGURE 3:**
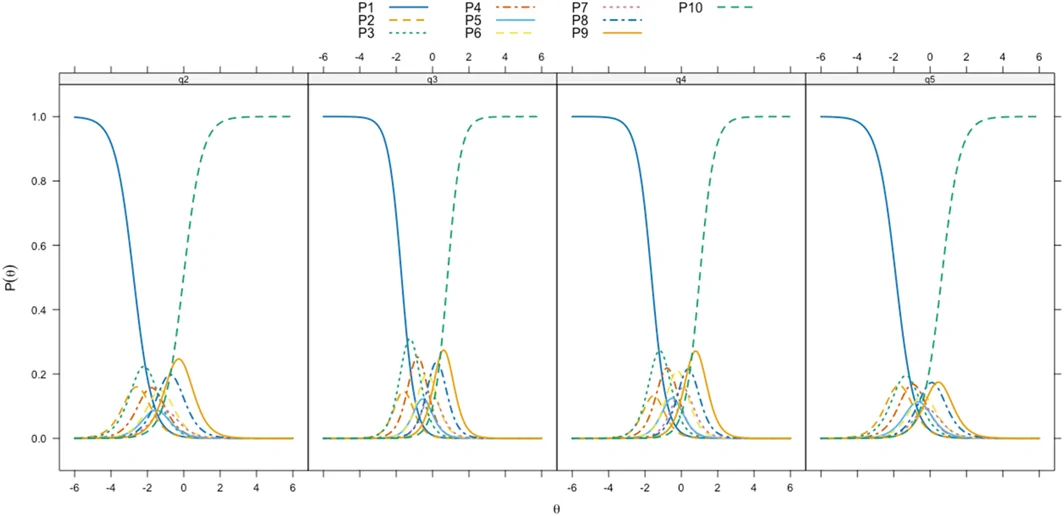
Item characteristic curves for the CWQ. Item characteristic (trace) curves from the graded response model illustrating the probability of endorsing each response category across levels of the latent trait. The ordered thresholds indicate appropriate functioning of the response categories for all items.

Item fit was evaluated using the S‐X^2^ statistic. Model fit was acceptable for all items.

The CWQ provided the greatest precision at low to moderate levels of the latent trait, with decreasing precision at higher levels (Figure 4).

**FIGURE 4:**
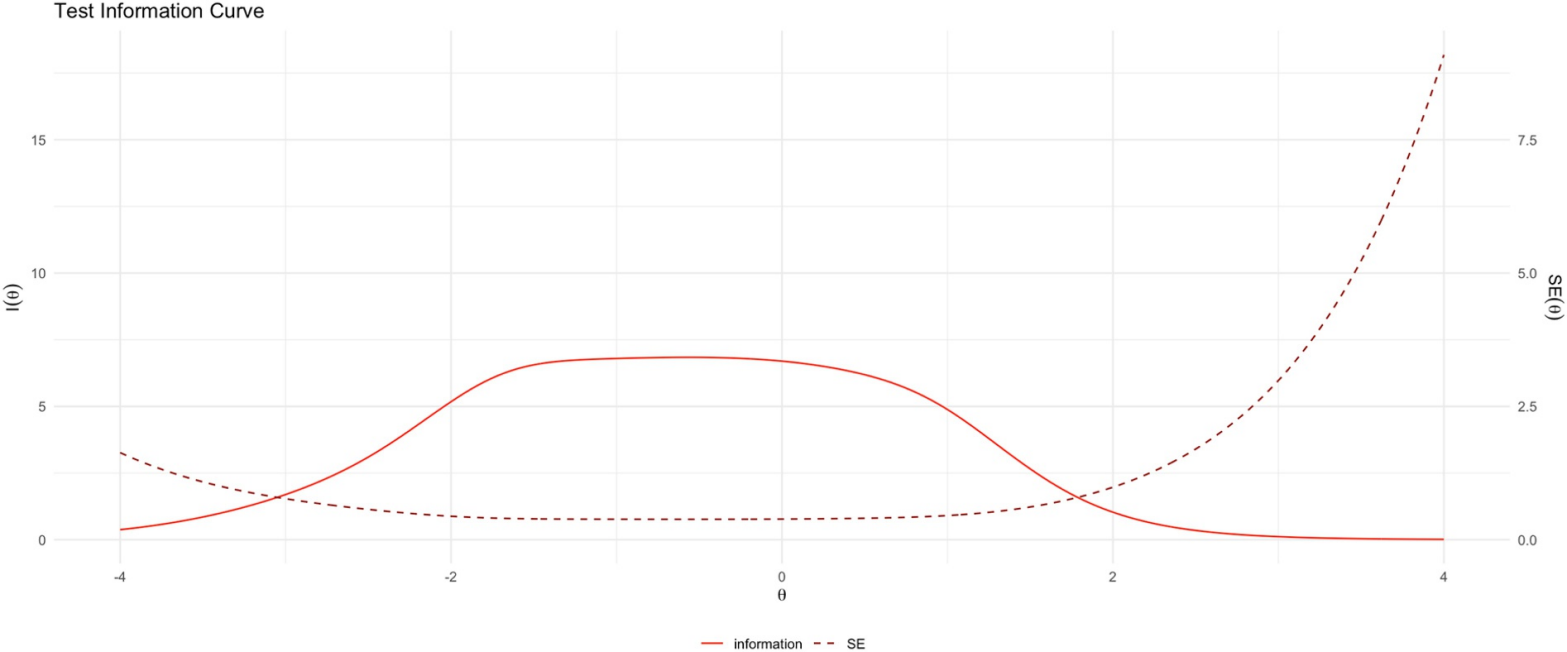
Test information and standard error of measurement for the CWQ. The CWQ shows greatest measurement precision at low to moderate levels of the latent trait.

Overall, the IRT findings supported the adequacy of the CWQ items and were consistent with results from the factor‐analytic and reliability analyses.

## 4 Discussion

### 4.1 Principal Findings

In this study, we evaluated the psychometric properties of the Swedish version of the Choosing Wisely Questions (CWQ) in a large psychiatric sample using both classical test theory and item response theory. We found evidence supporting the CWQ's structural validity, internal consistency, and item‐level performance. Overall, the results indicate that the CWQ provides a brief and psychometrically sound set of questions for assessing patients' perceptions of information given regarding medication treatment in psychiatry.

### 4.2 Psychometric Interpretation

Structural validity of the CWQ was supported by both exploratory and confirmatory factor analyses. Exploratory analyses demonstrated that a single factor accounted for a substantial proportion of variance, with all CWQ showing moderate to strong loadings. Higher values of CWQ total score were associated with endorsing higher responses on the items measuring participation, motivation and satisfaction with treatment, hence supporting convergent validity. Confirmatory factor analysis further supported a unidimensional structure. These findings justify the calculation and interpretation of a total CWQ score and indicate that the instrument measures a coherent underlying construct. Internal consistency of the CWQ was good, with Cronbach's alpha 0.81. This is in line with other shared decision‐making scales which have shown estimates of 0.68–0.93 (Ahmad et al. 2020). Given the brevity of the scale, this level of reliability suggests an appropriate balance between measurement precision and respondent burden. Findings from Mokken scale analysis strengthen confidence that higher CWQ scores reflect higher levels of the underlying construct. Finally, IRT analyses support the CWQ's intended use as a screening and evaluation tool rather than a diagnostic instrument. Taken together, the findings provide evidence for the CWQ's structural validity and internal consistency, fulfilling key COSMIN criteria for measurement quality.

### 4.3 Clinical Relevance

From a clinical perspective, the CWQ address a central component of patient‐centred care: patients' understanding of the benefits, risks, and alternatives of treatment options, and the implications of forgoing treatment (Elwyn et al. 2012). In psychiatric settings, where treatment decisions are often complex and preferences vary widely, structured tools that support shared decision‐making are particularly relevant. Recent research has revealed alarming reports of patients feeling excluded from decision‐making (Lindh et al. 2026), and both patients and clinicians have pointed to increased patient‐centered care being a prioritized research agenda (SBU 2026). A well‐informed patient is also necessary to avoid the low‐value care of prescribing medication that the patient might not want if they were properly informed about for example side‐effects.

### 4.4 Notes on the CWQ Scores

A concerning finding, however, was that patients felt more informed about the benefits of their medication, but less informed about risks, alternatives, and the implications of forgoing medication. This finding should urge improvement, since treatment decisions must be based on well‐informed patients being aware of both the benefits and risks. Within psychiatry, there are often alternatives, such as psychological or physical treatments, but also general psychosocial support, watchful waiting, psychoeducation, physical exercise, improved sleep, and so on. These might or might not outperform medications in clinical trials, but to practice true evidence‐based medicine, a clinician needs to discuss various alternatives according to the patients' values and preferences (Sackett et al. 1996).

### 4.5 Notes on Diagnoses

Some particularly interesting findings need to be addressed. The majority of patients had two or more ICD F‐diagnoses. Almost 9% had five or more and women tended to have more diagnoses; one had twelve. The conceptual problem of “comorbidity” in psychiatry has long been discussed (Nordgaard et al. 2023; van Loo and Romeijn 2015; Maj 2005). Issues regarding this heterogeneity of psychiatric diagnostic constructs have also been raised (Allsopp et al. 2019), and other approaches have been suggested to mitigate the effects (Cramer et al. 2010). Within psychiatric pharmacotherapy research, evidence is often derived from randomized controlled trials with strict inclusion and exclusion criteria where participants with “only” one diagnosis are included. However, since real‐world patients often have several psychiatric diagnoses and also somatic comorbidity (Plana‐Ripoll et al. 2019; Forslund et al. 2021; Taloyan et al. 2023), the evidence for drug treatment in these populations is weakened. These issues have been especially addressed in patients with, for example, borderline personality disorders (Hoertel et al. 2015), bipolar disorder (Wong et al. 2018) and depression (Zimmerman et al. 2019). Our results add evidence of multiple diagnoses being the norm within contemporary psychiatry. This is important when informing patients about treatment choices, since guidelines are often based on drug trials that might contain non‐representative populations.

### 4.6 Strengths and Limitations

Key strengths of this study include the large sample size, the use of multiple complementary psychometric methods, and adherence to COSMIN guidelines throughout the evaluation process. The inclusion of IRT analyses provides detailed insight into item functioning beyond traditional reliability estimates. Several limitations should also be acknowledged. The original response scale, with lower scores indicating more positive responses, may have been counter‐intuitive for participants and potentially increased the risk of response errors. Second, selection bias might be an issue with either very engaged or very discontented patients choosing to participate. However, active recruitment, as in our study, promotes efficacy (Liu et al. 2018). Also, we applied broad inclusion and exclusion criteria and participants were not selected based on their opinions about their treatment. But the overall proportion of registered patients who participated limit the generalizability of our findings, especially to more acutely ill or hospitalized psychiatric populations. Lastly, the marked variation in responses indicates the recruitment of participants with diverse opinions. Psychometrically, there are also some limitations. First, test–retest reliability was not assessed, as participants only completed the questionnaire at a single time point, limiting conclusions about the stability of scores over time, which is particularly important for clinical monitoring in psychiatric populations. Third, no criterion validity analyses were performed since there was no comparator. Fourth, although the psychiatric sample enhances relevance for mental health care, generalizability to other clinical populations remains to be established. Fifth, the CWQ comprises only four items. Including more items, such as the cost/insurance question omitted from the Swedish version, could broaden measurement precision, particularly at higher levels of the underlying latent trait. However, the few items may constitute a strength through mitigating response burden. Indeed, despite the few items, the scale shows good psychometric properties, which is a strength. Several scales within shared decision‐making have a similar number of items (Brodney et al. 2019).

### 4.7 Implications for Future Research

Future studies should examine the CWQ's test–retest reliability, responsiveness to change, and associations with external measures of shared decision‐making, treatment satisfaction, or clinical outcomes. Also, the CWQ's relation to other shared decision‐making scales (Ahmad et al. 2020) could clarify their utility. Validation in other clinical contexts and languages would further strengthen their applicability. Such work would support the CWQ's use both as a research instrument and for clinically monitoring and enhancing patient‐centred medication practices or health literacy. Their brevity facilitates their use in quality improvement initiatives and large‐scale evaluations of shared decision‐making.

## 5 Conclusion

Our study strengthens the psychometric validity of the CWQ and facilitates their implementation as an important patient‐reported outcome measure in psychiatric settings. Their use could advance patient‐centered care and shared decision‐making to reduce low‐value care. Clinicians should use them to inform their patients more thoroughly about all aspects that matter to them regarding their medication, thereby strengthening their autonomy and health literacy.

## Author Contributions

**Johan Bengtsson:** conceptualization, validation, writing – review and editing, project administration, supervision, resources, data curation, formal analysis, methodology, writing – original draft, funding acquisition. **Alma Andersson:** investigation, methodology, writing – original draft, writing – review and editing. **Natalie Forss Lilja:** investigation, methodology, writing – original draft, writing – review and editing. **Isabella Hultgren Farsani:** investigation, methodology, writing – original draft, writing – review and editing. **Ellen Lissbrant:** investigation, methodology, writing – original draft, writing – review and editing. **Bjorn Wettermark:** conceptualization, validation, project administration, supervision, resources, methodology, writing – review and editing. **Ida Blomqvist:** conceptualization, validation, visualization, writing – review and editing, project administration, supervision, data curation, formal analysis, methodology, software.

## Funding

This work was supported by regional grants from Region Uppsala (LUL‐1026675) and Fredrik and Ingrid Thuring's Foundation (2025‐210).

## Conflicts of Interest

The authors declare no conflicts of interest.

## Data Availability

Research data are not shared.
